# Supplementary material for: Tomato Twisted Leaf Virus: A Novel Indigenous New World Monopartite Begomovirus Infecting Tomato in Venezuela
Source: Viruses. 2019 Apr 4;11(4):327. doi: 10.3390/v11040327 (PMC6521247; doi:10.3390/v11040327)
Supplement: Supplementary file 1 [file viruses-11-00327-s001.pdf]

Communication

Tomato Twisted Leaf Virus: A Novel Indigenous New World Monopartite Begomovirus Infecting Tomato in Venezuela

Gustavo Romay [1,*], Francis Geraud-Pouey [2], Dorys T. Chirinos [3], Mathieu Mahillon [1], Annika Gillis [4,†], Jacques Mahillon [4] and Claude Bragard [1,*]

1 UCLouvain, Earth and Life Institute, Applied Microbiology-Phytopathology, Croix du Sud 2-L07.05.03, 1348 Louvain-la-Neuve, Belgium; mathieu.mahillon@uclouvain.be
2 La Universidad del Zulia (LUZ), Unidad Técnica Fitosanitaria, Maracaibo 4005, Estado Zulia, Venezuela; fgeraudp@gmail.com
3 Facultad de Ingeniería Agronómica, Universidad Técnica de Manabí, Manabí 130105, Ecuador; dtchirinos@gmail.com
4 UCLouvain, Earth and Life Institute, Applied Microbiology-Laboratory of Food and Environmental Microbiology, Croix du Sud 2-L7.05.12, 1348 Louvain-la-Neuve, Belgium; annika.gillis@uclouvain.be (A.G.); jacques.mahillon@uclouvain.be (J.M.)
† Present address: Section of Microbiology and Medical Research Council Centre for Molecular Bacteriology and Infection, Imperial College London, London SW72AZ, United Kingdom.
* Correspondence: gustavo.romay@uclouvain.be (G.R.); claude.bragard@uclouvain.be (C.B.)

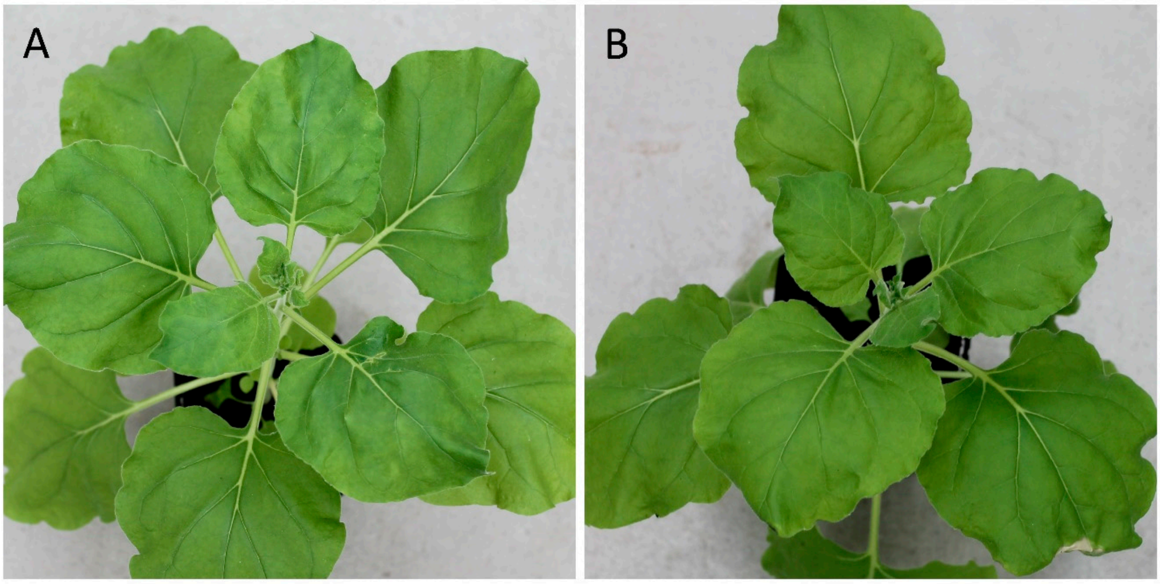

Figure S1. (A) *Nicotiana benthamiana* plant with mosaic symptoms at four weeks after agroinoculation with the ToTLV infectious clone. **(B)** *N. benthamiana* plant with no symptoms at four weeks after agroinoculation with the pCAMBIA1300 empty vector as a negative control
